# Computational analysis reveals the coupling between bistability and the sign of a feedback loop in a TGF-β1 activation model

**DOI:** 10.1186/s12918-017-0508-z

**Published:** 2017-12-21

**Authors:** Huipeng Li, Lakshmi Venkatraman, Balakrishnan Chakrapani Narmada, Jacob K. White, Hanry Yu, Lisa Tucker-Kellogg

**Affiliations:** 1 Computational and Systems Biology Program, Singapore-MIT Alliance, Singapore, 117576 Singapore; 2 Mechanobiology Institute, National University of Singapore, Singapore, 117411 Singapore; 3 NUS Graduate School for Integrative Sciences and Engineering, National University of Singapore, Singapore, 117456 Singapore; 4 Department of Physiology, National University of Singapore, Singapore, 117597 Singapore; 5 Department of Electrical Engineering and Computer Science, Massachusetts Institute of Technology, Cambridge, MA 02139 USA; 6 BioSystems and Micromechanics IRG, Singapore-MIT Alliance for Research and Technology, Singapore, 138602 Singapore; 7 Institute of Bioengineering and Nanotechnology, A*STAR, Singapore, 138669 Singapore; 8 Department of Biological Engineering, Massachusetts Institute of Technology, Cambridge, MA 02139 USA; 9 Center for Computational Biology, Duke-NUS Medical School, Singapore, 169857 Singapore; 10 Cancer and Stem Cell Biology, Duke-NUS Medical School, Singapore, 169857 Singapore

**Keywords:** Bistability, Positive feedback, Computational modelling, ODEs, Dynamical systems, Biochemical network, TGF-β1, Bifurcation analysis

## Abstract

**Background:**

Bistable behaviors are prevalent in cell signaling and can be modeled by ordinary differential equations (ODEs) with kinetic parameters. A bistable switch has recently been found to regulate the activation of transforming growth factor-β1 (TGF-β1) in the context of liver fibrosis, and an ordinary differential equation (ODE) model was published showing that the net activation of TGF-β1 depends on the balance between two antagonistic sub-pathways.

**Results:**

Through modeling the effects of perturbations that affect both sub-pathways, we revealed that bistability is coupled with the signs of feedback loops in the model. We extended the model to include calcium and Krüppel-like factor 2 (KLF2), both regulators of Thrombospondin-1 (TSP1) and Plasmin (PLS). Increased levels of extracellular calcium, which alters the TSP1-PLS balance, would cause high levels of TGF-β1, resembling a fibrotic state. KLF2, which suppresses production of TSP1 and plasminogen activator inhibitor-1 (PAI1), would eradicate bistability and preclude the fibrotic steady-state. Finally, the loop PLS − TGF-β1 − PAI1 had previously been reported as negative feedback, but the model suggested a stronger indirect effect of PLS down-regulating PAI1 to produce positive (double-negative) feedback in a fibrotic state. Further simulations showed that activation of KLF2 was able to restore negative feedback in the PLS − TGF-β1 − PAI1 loop.

**Conclusions:**

Using the TGF-β1 activation model as a case study, we showed that external factors such as calcium or KLF2 can induce or eradicate bistability, accompanied by a switch in the sign of a feedback loop (PLS − TGF-β1 − PAI1) in the model. The coupling between bistability and positive/negative feedback suggests an alternative way of characterizing a dynamical system and its biological implications.

**Electronic supplementary material:**

The online version of this article (10.1186/s12918-017-0508-z) contains supplementary material, which is available to authorized users.

## Background

**Fig. 1:**
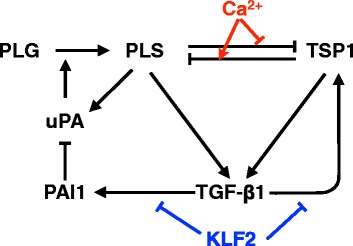
TGF-β1 bistable activation model. Black arrows represent the reactions from [5]. Red arrows represent the effects of calcium on the PLS-TSP1 interaction. Blue arrows represent the effects of KLF2 on PAI1 and TSP1 production. uPA is urokinase plasminogen activator, and PLG is plasminogen

Bistability has been found in many biological systems, and mathematical models using ordinary differential equations (ODEs) with respect to time provide a good representation for studying these dynamic behaviors [1–4]. Some bistable systems show a binary behavior at the level of single cells but exhibit a graded response for a population of cells [1], while other bistable systems cause a binary output even for populations of cells [5]. Different strategies have been developed to study the two different categories of bistable systems. For systems with bistability at the level of single cells, the bistable nature of the output is often well-known, guiding researchers to elucidate the underlying molecular circuit that enables the bistability. For systems with population-level bistability, the bistability may not be obvious by inspection, in which case, laborious experimental measurement is required to establish the phenomenon [5]. In a recent study, we modeled the bistable activation of transforming growth factor-β1 (TGF-β1), which belongs to the category of population-level bistability. The bistability was validated experimentally by showing hysteresis in an in vitro system [5]. TGF-β1 is a cytokine with broad importance for contexts such as cancer, liver cirrhosis, wound healing and regeneration. Our model captures multiple pathways with positive and negative effects towards TGF-β1 and its activators (Fig. 1; Additional file 1: Supplementary Note 1).

In the current study, we explore some possible triggers that could influence the bistable transition, and look for system properties that correlate with bistability. We demonstrate that factors like calcium and Krüppel-like factor 2 (KLF2) (Additional file 1: Supplementary Note 2) level can be modeled implicitly into the reaction rate parameters of the model, allowing us to discuss the on and off transitions of bistability under different conditions. We identified a coupling between the sign of a signaling loop in the model (i.e., whether the signaling loop shows positive feedback or negative feedback) and the presence of bistability in the model. This could suggest alternative ways to identify and validate of systems with population-level bistability.

## Results

### Calcium and KLF2 have potential influence on the steady state of TGF-β1 activation

#### Calcium would promote the steady state with high TGF-β1 activation

**Fig. 2:**
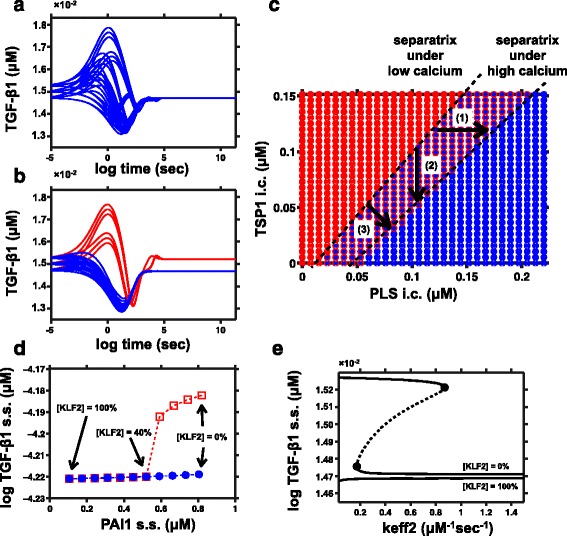
Calcium and KLF2 have potential influence on the steady state of TGF-β1 activation. **a**-**b** TGF-β1 abundance is plotted over time, after initializing the system from a given set of initial concentrations with (**a**) low calcium or (**b**) high calcium. In both cases, curves have been colored blue if they converge to a steady state with low TGF-β1 activation (ssP), and colored red if they converge to a steady state with high TGF-β1 activation (ssT). **c** Calcium causes a shift in the separatrix between steady states. Low-calcium and high-calcium simulations were performed using various initial concentrations of PLS and TSP1. After observing in Fig. 2a-b that initial conditions with 0.5ssP and 0.5ssT were usually in the basin of convergence for the ssP state (for the low calcium model), we decided to shift the initial conditions. For studying the behavior of the separatrix, the initial concentrations were set to .25*ssP and .75*ssT. The steady state outcomes are shown by colors, with red indicating the steady state with high TGF-β1 (ssT), and blue indicating low TGF-β1 (ssP). For each combination of PLS and TSP1, the low calcium result is indicated by the color of the small inner dot, and the high-calcium result is indicated by the color of the outer circle. **d** Two steady state (s.s.) levels of TGF-β1 and PAI1 under different levels of KLF2. Red squares represent steady states with high TGF-β1 activation (ssT), while blue dots represent steady states with low TGF-β1 activation (ssP). The y-axis is the log of the steady state concentration of TGF-β1. **e** Bifurcation analysis under KLF2 low and KLF2 high conditions

We built the low and high calcium variants of the model by considering the potential effects of calcium on the PLS-TSP1 interaction (see Methods, Fig. 1 red arrows). Three parameters for the calcium effect were not known quantitatively and were estimated (see Methods). In Fig. 2a-b, we simulated the low calcium model and the high calcium model over time with 27 total initial configurations. These 27 configurations were combinations of 3 initial concentrations for each of TGF-β1, TSP1, and plasmin, the ssT level, ssP level, and the mean level of ssT and ssP (27 = 3^3^). The initial concentrations of other species were set to the average of their two steady state levels, (i.e., 0.5ssT + 0.5ssP). In the low-calcium model, all trajectories converged to ssP with low TGF-β1, but in the high-calcium model, several of the initial configurations converged to ssT with high TGF-β1. To generalize our understanding of this effect, we plotted the boundary (the separatrix, Fig. 2c) between the initial configurations that caused convergence toward ssT (red) and the initial conditions that caused convergence toward ssP (blue). Initial concentrations were constants for all species other than PLS and TSP1. By comparing the separatrix of the low calcium model (dot) and the high calcium model (circle), we observe a shift of the separatrix toward the blue (ssP) region. This means the red (ssT) region is enlarged in high calcium environment (arrow 3). As expected, calcium tips the balance between PLS and TSP1 to achieve a significant effect on steady state of TGF-β1 activation.

#### KLF2 would eliminate the steady state with high TGF-β1 activation

KLF2 is a transcription factor studied extensively in atherosclerosis and fibrosis, and previous studies of KLF2 signaling showed TSP1 and PAI1 (plasminogen activator inhibitor-1) to be two of its most strongly affected targets [6, 7]. To study how KLF2 would affect bistable activation of TGF-β1, variants of the TGF-β1 activation model were built as described in methods (Fig. 1 blue arrows). We built a model called “100% KLF2” that downregulated the TSP1 production and PAI1 production rates, proportional to the published effects of KLF2 on the mRNA levels of TSP1 (−7.8 fold) and PAI1 (−7.4 fold). This is a strong effect, so we also built models with 90%, 80%, … 10%, and 0% of the KLF2 effects on the TSP1 and the PAI1 production rates. Each model in the series was simulated to obtain the steady state concentrations. When a dynamical system is bistable, its two steady states are commonly obtained by simulating the model twice, once starting from each side of the separatrix boundary (for example, initializing the system with opposite extreme levels of TGF-β1). For our series of models, the steady states obtained after initialization with high TGF-β1 (resembling ssT) were plotted with open red boxes, and the steady states obtained after initialization with low TGF-β1 (resembling ssP) were plotted with solid blue circles (Fig. 2d). Models for each level of KLF2 were plotted in terms of PAI1 and TGF-β1 steady states (with KLF2 levels decreasing from left to right). For models with KLF2 ≥ 40%, the open red boxes fell at the same points as the solid blue circles, indicating they are monostable. For models with KLF2 ≤ 30%, the red open boxes were distinct from the blue circles, indicating two steady states. For KLF2 levels from 0% to 100% (right to left), the low TGF-β1 steady state (blue dots) remained almost constant, while the high TGF-β1 steady state (red boxes) merged with the low TGF-β1 steady state in an ultrasensitive manner when the KLF2 effect increased from 30% to 40%.

Bifurcation analysis studies how parameter change affects the qualitative behavior and the steady states of a system [8]. Bifurcation plot allows us to see all the equilibria of the system and how the equilibria vary with change of KLF2 levels and other rate parameters. We chose one parameter named “keff2” to show the steady state behavior of the low KLF2 and high KLF2 system. “Keff2”, the enzymatic efficiency of plasmin, is one of many rate parameters that affect the overall bistability of the system. Figure 2e shows the bifurcation plot for the 0% KLF2 model and the 100% KLF2 model with respect to the parameter “keff2”. Solids lines represent the stable steady states of the system (ssP or ssT). Dotted lines represent one unstable steady state between the two stable steady states, which is not achievable through simulation. Black circles represent two limit points in the bifurcation curve, which separates the monostable regime and bistable regime. Bifurcation analysis confirmed that the system with 0% KLF2 retained bistability (an “S-shaped” curve in Fig. 2e) while the system with 100% KLF2 was monostable.

### The bistability of the system correlates with the sign of the PLS-PAI1 feedback loop

**Fig. 3:**
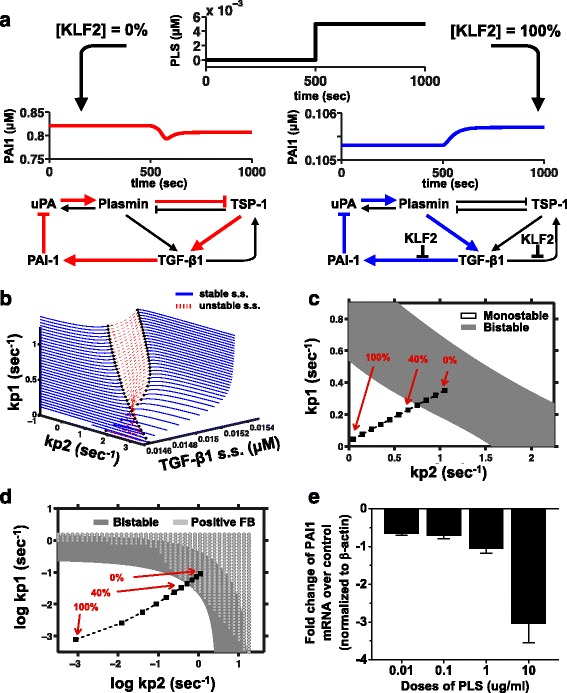
The bistability of the system correlates with the sign of the PLS-PAI1 feedback loop. **a** KLF2 affects the sign of PLS-PAI1 feedback loop. We designed an exogenous addition of PLS into the system using a step function for the level of PLS over time (top panel, black curve). Stimulating the TGF-β1 activation model with exogenous PLS caused two different effects in silico, depending on the KLF2 status. In the absence of KLF2 (red curve on left), the stimulus caused positive (double-negative) feedback between PAI1 and PLS, which can occur via the red arrows shown. In the presence of KLF2 (blue curve on right), exogenous PLS treatment caused a positive effect on PAI1 and the negative feedback loop (blue arrows) was restored. **b** Methods to calculate the 2d bistable region. We did bifurcation analysis of *kp2* for a series of *kp1* value (100 equally spaced values between *kp1* = 1.2 and *kp1* = 0) for the TGF-β1 activation model. The stable s.s. on the bifurcation curve were plotted as solid blue line, while the unstable s.s. were plotted as dotted red line. The Limit Points (LP), which tell the bistable interval of the bifurcation parameter (*kp2*) were denoted by black dots on the bifurcation curve. **c** The series of KLF2 levels cross the boundary of the bistable region in *kp1*-*kp2* phase plane. The 2d bistable region (dark gray) in kp1-kp1 phase plane can be constructed based on the kp2 coordinates of LPs in (**b**) and their corresponding *kp1* values. KLF2 levels are represented as diamond dots. **d** Overlap between bistable region and PLS-PAI1 positive feedback region in the *kp1*-*kp2* phase plane. Bistable region is denoted by solid dark gray, while PLS-PAI1 positive feedback region is denoted by dotted light gray. The x and y axis are all in log scale. **e** Empirically, PLS can down-regulate PAI1 gene expression in a co-culture of hepatocytes and HSC-T6 cells. Hepatic stellate cells (HSC-T6) were cultured with primary rat hepatocytes in a 7:1 ratio overnight, creating a fibrosis-like state with high TGF-β1 level. The next day, the medium was changed to non-serum medium with different doses of PLS. Cells were collected 24 h later

Positive and negative feedback loops are ubiquitous in biological systems [9], and necessary for many functions [10, 11]. The TGF-β1 activation network is composed of multiple overlapping feedback loops, including two feedback loops between PLS and PAI1. One obvious loop is the negative feedback loop PLS → TGF-β1 → PAI1⊣PLS, which is frequently cited [12–16]. A less obvious loop is PLS⊣TSP1 → TGF-β1 → PAI1⊣PLS, with two inhibitory effects, meaning positive feedback (See Fig. 3. a-b). Interestingly, experiments have already observed two opposite behaviors of PLS towards TGF-β1 and PAI1 [17–19], giving indirect evidence for the possibility of both positive and negative feedback loops involving PLS, TGF-β1, and PAI1.

To characterize the feedback between PLS and PAI1 in this network, we plotted feedback behavior in both low KLF2 (0%) and high KLF2 (100%) models (Fig. 3a). Since PAI1 (plasminogen activator inhibitor-1) is antagonistic towards PLS, the sign of the PLS-PAI1 feedback loop is determined by the response of PAI1 to PLS. We used a stepwise PLS input (black curve) to perturb both the low (%0) KLF2 model and the high (100%) KLF2 model and we simulated the response of PAI1. The low KLF2 model showed a decrease in the level of PAI1, which means that the overall PLS-PAI1 feedback is dominated by the PLS⊣TSP1 → TGF-β1 → PAI1⊣PLS double negative (positive) feedback loop. The high KLF2 model showed an increase in the level of PAI1, which means that the overall PLS-PAI1 feedback is dominated by the PLS → TGF-β1 → PAI1⊣PLS negative feedback loop.

It was interesting to observe in simulation that KLF2 not only was able to eliminate one of the steady states and turned the system into monostable, but also able to change the sign of PLS-PAI1 feedback loop. To further characterize the effect of KLF2 on the system and discuss the reason behind it, we plotted the 2d bistable region of the system in the *kp1*-*kp2* phase plane. *kp1* is the TSP1 synthesis rate parameter and *kp2* is the PAI1 synthesis parameter, both of which affect the bistability of the system. We found the boundaries of the bistable region through equilibrium continuation of *kp2* for a series of *kp1* values (Fig. 3b). In our model, KLF2 is represented as a combination of fold changes of *kp1* and *kp2*, therefore, KLF2 levels can be represented as a series of points in the *kp1*-*kp2* phase plane (Fig. 3c). It can be seen on Fig. 3c that KLF2 point is moving out of the bistable region when KLF2 increases from 0 to 100%. We then analyzed the sign of PLS-PAI1 feedback in this *kp1*-*kp2* phase plane. Interestingly, there is a large overlap between the bistable region of the system and positive feedback region of PLS-PAI1 feedback loop (Fig. 3d). KLF2 = 0 point lies in the overlapping area of the bistable region and PLS-PAI1 positive feedback region, while KLF2 = 100% lies in the overlapping area of the non-bistable region and PLS-PAI1 negative feedback region. This explains why the change of KLF2 level can have two different effects on the system.

The large overlap between bistable region and PLS-PAI1 positive feedback region is also an interesting property of the system, since it suggests that in reality, a bistable TGF-β1 activation system most likely also has positive PLS-PAI1 feedback. Although negative feedback loop between PLS-PAI1 has been observed repeatedly and is well accepted [17, 18], positive feedback would be novel. We tested the sign of the feedback from PLS to PAI1 using an experimental system known to exhibit TGF-β1 bistability [5], a cell culture model of liver fibrosis. In this co-culture with primary hepatocytes and HSC-T6 cell lines, we added different levels of PLS and we measured PAI1 mRNA levels using RT-PCR (Fig. 3b). Increasing PLS was found to cause decreased expression of PAI1 in this bistable system, implying that PLS and PAI1 can indeed exhibit positive feedback.

## Discussion

Using a model of TGF-β1 activation, we explored the ability of external factors to switch bistability on and off, and we characterized the correlation between the bistability of the model, and the sign of a feedback loop in the network.

For the first part, we modeled known effects of calcium on the balance between TSP1 and PLS [20–25], and known effects of KLF2 on the gene expression of PAI1 and TSP1 [6]. We then used modeling to show how these effects would propagate through the system. Specifically, the model predicted that calcium would significantly promote TGF-β1 activation, shifting the bistable threshold of the system. The calcium-induced increase in TSP1 would lie within the physiological range of TSP1 [26]. Literature search reveals that extracellular calcium may be relatively easy to perturb via biomaterials of bandages, etc. Therefore, the effect of extracellular calcium on TGF-β1 might have important therapeutic implications for fibrotic or inflammatory diseases where abnormal TGF-β1 contributes to disease. For example, fibrotic diseases are driven by high levels of TGF-β1 [27], and therapeutic studies in animals have achieved significant access by increasing the PLS pathway [28] or decreasing the TSP1 pathway [29]. In our model, if we take the ssP state to be healthy and the ssT state to be fibrotic, then a fibrotic system with high calcium could transition toward health through an increase of PLS (Fig. 2c, arrow 1), through a decrease of TSP1 (Fig. 2c, arrow 2), or a combination of both (Fig. 2c, arrow 3).

In contrast, KLF2 was simulated to increase PLS activity and decrease the levels of TGF-β1, by suppressing PAI1 and TSP1 expression. This is consistent with previous work with statin drugs on liver fibrosis [30], where KLF2 upregulation was observed after treatment with simvastatin. Our model predicts that one of the ways KLF2 may contribute to improvement of liver fibrosis may be by decreasing the activation of TGF-β1 through reduction of the TSP1 and PAI feedback effects.

While modeling KLF2 effects, we noticed that loss of bistability also caused a change in the sign of the PLS - PAI1 feedback loop. Without KLF2, the PLS-PAI1 feedback loop was positive (double negative), but with KLF2 (100% KLF2) and with the destruction of bistability, the PLS-PAI1 feedback loop was negative. Additional bifurcation analysis revealed that high KLF2 is a special case of the general observation, that the bistability of the system is correlated with the sign of the PLS-PAI1 feedback loop.

We demonstrated the positive feedback behavior between PLS and PAI1 in the bistable TGF-β1 system, using an in vitro experiment. Previous studies have already revealed two opposite behaviors of PLS towards TGF-β1 and PAI1 [17–19], providing indirect evidence for the possibility of both positive and negative feedback loops. Some aspects of the feedback loop are relatively unambiguous. For example, PAI1 is a specific and potent inhibitor of plasmin activation. PAI1 production follows TGF-β1 signaling so closely that, in practice, PAI1 levels are commonly measured as a readout of TGF-β1 activation [31]. The behavior of the feedback loop thus boils down to the behavior of the PLS – TGF-β1 relationship. In isolation, PLS clearly is able to activate TGF-β1. The same effect has frequently been observed in more physiological contexts, and there is considerable published evidence that PLS and/or plasminogen activators can cause an increase in TGF-β1 and/or PAI1 levels [12–16]. This positive effect of PLS on TGF-β1 or PAI1 serves as evidence that the loop between PLS and PAI1 can have negative feedback. Although the activating ability of PLS toward TGF-β1 is well known and accepted, some studies also suggest the opposite effect. For example, PLS caused TGF-β2 levels to decline in breast cysts [32]. Furthermore, one unconventional finding by Seo et al., showed a positive feedback effect between PAI1 and TGF-β1 [19], suggesting that PLS can cause a decrease in TGF-β1. In sum, we found that there is some support in the published literature for our prediction that the relationship between PLS and PAI can show either negative feedback or positive feedback, depending on context.

Another important consideration in interpreting this model is the redundancy of proteases and matrix factors that play roles similar to PLS or TSP1. Actually, PLS is only one of many proteases (including elastase, MMP-2, MMP-9, ADAMTS1 and others) that can both activate TGF-β1 and cleave TSP1 [33–36]. Meanwhile TSP1 can inhibit many of these proteases [20, 37–39]. Extracellular proteases often function interdependently by activating each other (e.g. PLS activates several MMPs, which activate other MMPs [40, 41]), and some proteases may have partially redundant effects. Likewise TSP1 may represent a larger class of matrix proteins and mechanical factors with redundant roles in this model. Fibrillin and LTBP1 can promote TGF-β1 activation [42–45], as can factors that create mechanical tension in the matrix [46, 47]. Fibrillin and LTBP1 can be cleaved by PLS [14, 48], and even mechanical tension would be antagonized by PLS cleavage. In other words, PLS and TSP1 are archetypes of two larger classes of effects, a protease category and a matrix category, that may be capable of antagonizing the effect of each other, even as they contribute individually to TGF-β1 activation. The redundancy of the protease-versus-matrix competition suggests that this antagonism may be an organizing principle of TGF-β1 regulation, with evolutionary importance to the organism. On the other hand, this redundancy also creates many complexities that could perturb the phenomena we simulated. For example, the effects we attribute to PLS itself may actually result from the indirect effects of PLS-activated proteases. Thus, we speculate that the insights we drew from this model point to important properties of general TGF-β1 activation regulatory networks in different contexts.

## Conclusions

Using TGF-β as a case study, we demonstrated that external factors could influence the bistability of the model, and that these influences can be modeled implicitly using the reaction parameters of the model. Furthermore, we showed that system-level properties like the sign of feedback loops can correlate with the bistability of a complex model. This provides a novel characterization of the transition between bistable and monostable regimes, and provides a non-obvious explanation for seemingly contradictory experimental findings about the contribution of the PAI1 - PLS feedback loop toward TGF-β activation.

## Methods

### TGF-β1 bistable activation model

We used the model built by Venkatraman et al. as the base model of TGF-β1 regulation. We increased the “k_others_” parameter relative to the published model, to allow for higher basal activation of TGF-β1 by other activators such as integrins [42, 49, 50]. Simulations were performed using kroneckerbio toolbox [51] and the ode15s solver in MATLAB (Mathworks, Natick, MA).

### Calcium model

Calcium can affect the structure of TSP1 [22–25], the enzymatic activity of PLS cleaving TSP1 [20], and the ability of TSP1 to inhibit serine protease activity [21]. These effects were represented by the rate constants k3, k_3 and k4 in the TGF-β1 activation model. We used the original parameter settings as the low calcium settings. To reflect high calcium conditions, we increased k3 by 10 fold, decreased k_3 by 0.1 fold, and k4 by 0.0001 fold, in order to reflect a high level of calcium in the environment. Details of the model can be found in Table S1 (Additional file 2) and Table S2 (Additional file 3).

### KLF2 model

It has been shown that KLF2 can decrease TSP1 expression by 7.8 fold and PAI1 expression by 7.4 fold [6, 7]. We simulated the TGF-β1 activation model with no change (0% of the KLF2 effect, original parameter settings), with 100% of the KLF2 effect (7.4 fold decrease of PAI1 synthesis parameter *kp2* and 7.8 fold decrease of TSP1 synthesis parameter *kp1*), as well as a series of intermediate models with 10%, 20%, … 90% of the KLF2 effect, causing intermediate levels of decrease in the PAI1 and TSP1 synthesis rates.

### Bifurcation analysis

Bifurcation analysis was performed using MATCONT (https://sourceforge.net/projects/matcont/). Equilibrium continuation function was called to generate the bifurcation curves in Fig. 2e and Fig. 3b.

### Experimental methods

Isolation of primary hepatocytes was performed on male Wistar rats (250-300 g), via a two-step collagenase perfusion method as described previously [52]. A co-culture model of primary rat hepatocytes and hepatic stellate cell line T6 (HSC-T6) was established as described in [5]. Briefly, primary rat hepatocytes were first seeded at a density of 2 × 10^5^ cells on 35 mm collagen-coated dishes (IWAKI) using Williams’s E media with 10% FBS. After 4 h, hepatic stellate cell line T6 (HSC-T6) was seeded at a density of 1.4 × 10^6^ cells. The cells were cultured overnight in 35 °C, and 5% CO_2_ in William’s E media with 2% FBS to facilitate HSC activation. The next day media was changed to Williams’s E without serum, along with different doses of PLS. After 24 h, the cells were collected.

RT-PCR was performed as described in [53]. Briefly, mRNA was isolated from the cells using RNeasy mini kit (Qiagen), and its concentration was quantified using a Nanodrop 2000 UV-Vis Spectrophotometer. One microgram of mRNA from each sample was converted to cDNA (Invitrogen, Superscript Reverse Transcriptase III) and real-time PCR reaction (Roche, Sybr Green Master mix) was carried out for plasminogen activator inhibitor-1 (PAI1) and β-actin, with in-house primers shown in Table S3 (Additional file 4). The gene expression values were determined by the Del-Del C_T_ relative quantitation method; the target C_T_ values were normalized to the endogeneous reference β-actin, and the normalized mRNA was expressed as a fold-change relative to the untreated control.

## Additional files

Additional file 1: Supplementary Notes. (PDF 107 kb)

Additional file 2: Table S1. List of equations and parameters used for model construction. (PDF 280 kb)

Additional file 3: Table S2. Parameters settings for different models. (PDF 47 kb)

Additional file 4: Table S3. List of primer sequences for genes probed on quantitative real time PCR. (PDF 26 kb)

## Abbreviations

PAI1: Plasminogen activator inhibitor-1
PLG: Plasminogen
PLS: Plasmin
ssP: PLS predominant steady state
ssT: TSP1 predominant steady state
TGF-β1: Transforming growth factor β1
TSP1: Thrombospondin-1
