# Supplementary material for: Computational analysis reveals the coupling between bistability and the sign of a feedback loop in a TGF-β1 activation model
Source: BMC Syst Biol. 2017 Dec 21;11(Suppl 7):136. doi: 10.1186/s12918-017-0508-z (PMC5763301; doi:10.1186/s12918-017-0508-z)
Supplement: Supplementary file 1 — Supplementary Notes. (PDF 107 kb) [file 12918_2017_508_MOESM1_ESM.pdf]

Supplementary Notes:

1: Components of TGF-β1 bistable mode

The full model is described in Fig. 1 (Fig. 1, black arrows). To summarize this model, TGF-β1 is activated by plasmin (PLS) or thrombospondin-1 (TSP1), each having its own feedback mechanisms [1]. PLS feedback occurs because PLS activates TGF-β1 [2, 3], and then TGF-β1 upregulates plasminogen activator inhibitor-1 (PAI1), which is an inhibitor of plasminogen activation [4, 5]. These effects form a negative feedback loop between TGF-β1 and PLS. In contrast, TSP1 feedback occurs because TSP1 activates TGF-β1, and TGF-β1 upregulates TSP1 [6-8], creating a positive feedback loop between TGF-β1 and TSP1. There is also mutual antagonism between PLS and TSP1 [9, 10], which pushes the system towards either a "PLS-predominant" steady state, or "TSP1-predominant" steady state. The "PLS-predominant" steady state (ssP) has moderate levels of TGF-β1 due to negative feedback [1]. In contrast, the TSP1-predominant steady state (ssT) achieves a higher level of TGF-β1 activation due to positive feedback [1]. Venkatraman et al. have shown that introducing extra PLS into a TSP1 dominant system would switch the balance between PLS and TSP1, thus inducing a decrease in the level of active TGF-β1.

2: Calcium and KLF2 as regulators of TGF-β1 activity

Literature search yielded two factors that could regulate both PLS and TSP1, calcium [11, 12] and Krüppel-like factor 2 (KLF2) [13-15]. Calcium has been found to enhance the inhibition of the activity of PLS by TSP1, and suppress the cleavage of TSP1 by PLS

[11, 12]. KLF2 have been found to cause significant suppression of TSP1 and PAI1 [14, 15], meaning that KLF2 could regulate both PLS and TSP1 sub-pathways of TGF-β1 activation.

References:

1. Venkatraman L, Chia S-M, Narmada BC, White JK, Bhowmick SS, Forbes Dewey C Jr., So PT, Tucker-Kellogg L, Yu H: **Plasmin Triggers a Switch-Like Decrease in Thrombospondin-Dependent Activation of TGF-β1**. *Biophys. J.* 2012, **103**:1060–1068.

2. Annes JP, Munger JS, Rifkin DB: **Making sense of latent TGFbeta activation.** *J. Cell. Sci.* 2003, **116**:217–224.

3. Lyons RM, Gentry LE, Purchio AF, Moses HL: **Mechanism of activation of latent recombinant transforming growth factor beta 1 by plasmin.** *J. Cell Biol.* 1990, **110**:1361–1367.

4. Kutz SM, Hordines J, McKeown-Longo PJ, Higgins PJ: **TGF-beta1-induced PAI-1 gene expression requires MEK activity and cell-to-substrate adhesion.** *J. Cell. Sci.* 2001, **114**:3905–3914.

5. Thorsen S, Philips M, Selmer J, Lecander I, Astedt B: **Kinetics of inhibition of tissue-type and urokinase-type plasminogen activator by plasminogen-activator inhibitor type 1 and type 2.** *Eur. J. Biochem.* 1988, **175**:33–39.

6. Sajid M, Lele M, Stouffer GA: **Autocrine thrombospondin partially mediates TGF-beta1- induced proliferation of vascular smooth muscle cells.** *Am. J. Physiol. Heart Circ. Physiol.* 2000, **279**:H2159–65.

7. Mimura Y, Ihn H, Jinnin M, Asano Y, Yamane K, Tamaki K: **Constitutive thrombospondin-1 overexpression contributes to autocrine transforming growth factor-beta signaling in cultured scleroderma fibroblasts.** *Am. J. Pathol.* 2005, **166**:1451–1463.

8. Murphy-Ullrich JE, Poczatek M: **Activation of latent TGF-beta by thrombospondin-1: mechanisms and physiology.** *Cytokine Growth Factor Rev.* 2000, **11**:59–69.

9. Bonnefoy A, Legrand C: **Proteolysis of subendothelial adhesive glycoproteins (fibronectin, thrombospondin, and von Willebrand factor) by plasmin, leukocyte cathepsin G, and elastase.** *Thromb. Res.* 2000, **98**:323–332.

10. Hogg PJ, Stenflo J, Mosher DF: **Thrombospondin is a slow tight-binding inhibitor of plasmin.** *Biochemistry* 1992, **31**:265–269.

11. Anonick PK, Yoo JK, Webb DJ, Gonias SL: **Characterization of the**

antiplasmin activity of human thrombospondin-1 in solution. *Biochem. J.* 1993, **289 (Pt 3)**:903–909.

12. Gotis-Graham I, Hogg PJ, McNeil HP: **Significant correlation between thrombospondin 1 and serine proteinase expression in rheumatoid synovium.** *Arthritis Rheum.* 1997, **40**:1780–1787.

13. Marrone G, Russo L, Rosado E, Hide D, García-Cardeña G, García-Pagán JC, Bosch J, Gracia-Sancho J: **The transcription factor KLF2 mediates hepatic endothelial protection and paracrine endothelial-stellate cell deactivation induced by statins.** *J. Hepatol.* 2012.

14. Dekker RJ: **KLF2 provokes a gene expression pattern that establishes functional quiescent differentiation of the endothelium**. *Blood* 2006, **107**:4354–4363.

15. Boon RA, Fledderus JO, Volger OL, van Wanrooij EJA, Pardali E, Weesie F, Kuiper J, Pannekoek H, Dijke Ten P, Horrevoets AJG: **KLF2 suppresses TGF-beta signaling in endothelium through induction of Smad7 and inhibition of AP-1.** *Arterioscler. Thromb. Vasc. Biol.* 2007, **27**:532–539.
