# Supplementary material for: Computational analysis reveals the coupling between bistability and the sign of a feedback loop in a TGF-β1 activation model
Source: BMC Syst Biol. 2017 Dec 21;11(Suppl 7):136. doi: 10.1186/s12918-017-0508-z (PMC5763301; doi:10.1186/s12918-017-0508-z)
Supplement: Supplementary file 2 — List of equations and parameters used for model construction. (PDF 280 kb) [file 12918_2017_508_MOESM2_ESM.pdf]

TABLE S1 List of equations and parameters used for model construction

Reaction equation	Reaction equation
$scUPA + PLG \xrightarrow{keff_1} PLS + scUPA$	$A2M + PLS \underset{k_{-5}}{\overset{k_5}{\rightleftarrows}} A2M : PLS$
$PLS + scUPA \xrightarrow{keff_2} tcUPA + PLS$	$PAI1 + tcUPA \underset{k_{-6}}{\overset{k_6}{\rightleftarrows}} PAI1 : tcUPA$
$tcUPA + PLG \xrightarrow{keff_3} PLS + tcUPA$	$PAI1 + scUPA \underset{k7}{\overset{k_7}{\rightleftarrows}} PAI1 : scUPA$
$PLS + LTGF\beta1 \xrightarrow{k_1} TGF\beta1 + PLS$	$PLS + scUPA \xrightarrow{keff_2} tcUPA + PLS$
$TSP1 + LTGF\beta1 \xrightarrow{k_2} TGF\beta1 + PLS$	$PLS + scUPA \xrightarrow{keff_2} tcUPA + PLS$
$LTGF\beta1 \xrightarrow{k_{others}} TGF\beta1$	$TSP1 : PLS \xrightarrow{k_8}$
$TGF\beta1 \xrightarrow{kp_1} TSP1$	$TGF\beta1 \xrightarrow{k_9}$
$TGF\beta1 \xrightarrow{kp_2} PAI1$	$\xrightarrow{\alpha_1} \{scUPA;\ LTGF\beta1;\ A2M\};\quad \xrightarrow{\alpha_2} \{PLG\}$
$TSP1 + PLS \underset{k_{-3}}{\overset{k_3}{\rightleftarrows}} TSP1 : PLS$	$\{scUPA;\ LTGF\beta1;\ A2M\} \xrightarrow{\mu_{e\deg}};$ $\{\text{all other protein species}\} \xrightarrow{\mu_{p\deg}}$
$TSP1 : PLS \xrightarrow{k_4} PLS$	

Reactions of TGF-β1 activation model as described in [1]. Ordinary differential equations are generated from these reactions using mass-action law.
