# Supplementary material for: Computational analysis reveals the coupling between bistability and the sign of a feedback loop in a TGF-β1 activation model
Source: BMC Syst Biol. 2017 Dec 21;11(Suppl 7):136. doi: 10.1186/s12918-017-0508-z (PMC5763301; doi:10.1186/s12918-017-0508-z)
Supplement: Supplementary file 3 — Parameters settings for different models. (PDF 47 kb) [file 12918_2017_508_MOESM3_ESM.pdf]

TABLE S2 Parameters settings for different models

Parameters	low calcium, [KLF2] = 0%	high calcium, [KLF2] = 0%	low calcium [KLF2] = 100%
$keff_1$	0.035 $\mu M^{-1}s^{-1}$	0.035 $\mu M^{-1}s^{-1}$	0.035 $\mu M^{-1}s^{-1}$
$keff_2$	0.35 $\mu M^{-1}s^{-1}$	0.35 $\mu M^{-1}s^{-1}$	0.35 $\mu M^{-1}s^{-1}$
$keff_3$	1.4 $\mu M^{-1}s^{-1}$	1.4 $\mu M^{-1}s^{-1}$	1.4 $\mu M^{-1}s^{-1}$
k_1	0.035 $\mu M^{-1}s^{-1}$	0.035 $\mu M^{-1}s^{-1}$	0.035 $\mu M^{-1}s^{-1}$
k_2	24.5 $\mu M^{-1}s^{-1}$	24.5 $\mu M^{-1}s^{-1}$	24.5 $\mu M^{-1}s^{-1}$
k_{others}	0.35 s^{-1}	0.35 s^{-1}	0.35 s^{-1}
kp_1	0.35 s^{-1}	0.35 s^{-1}	0.35/7.8 s^{-1}
kp_2	1.05 s^{-1}	1.05 s^{-1}	1.05/7.4 s^{-1}
k_3	17.5 $\mu M^{-1}s^{-1}$	175 $\mu M^{-1}s^{-1}$	17.5 $\mu M^{-1}s^{-1}$
k_{-3}	0.0245 s^{-1}	0.00245 s^{-1}	0.0245 s^{-1}
k_4	0.35 $\mu M^{-1}s^{-1}$	3.5×10^{-5} $\mu M^{-1}s^{-1}$	0.35 $\mu M^{-1}s^{-1}$
k_5	24.5 $\mu M^{-1}s^{-1}$	24.5 $\mu M^{-1}s^{-1}$	24.5 $\mu M^{-1}s^{-1}$
k_{-5}	0.0105 s^{-1}	0.0105 s^{-1}	0.0105 s^{-1}
k_6	0.035 $\mu M^{-1}s^{-1}$	0.035 $\mu M^{-1}s^{-1}$	0.035 $\mu M^{-1}s^{-1}$
k_{-6}	0.0035 s^{-1}	0.0035 s^{-1}	0.0035 s^{-1}
k_7	0.07 $\mu M^{-1}s^{-1}$	0.07 $\mu M^{-1}s^{-1}$	0.07 $\mu M^{-1}s^{-1}$
k_{-7}	0.0035 s^{-1}	0.0035 s^{-1}	0.0035 s^{-1}
k_8	24.5 s^{-1}	24.5 s^{-1}	24.5 s^{-1}
k_9	0.21 s^{-1}	0.21 s^{-1}	0.21 s^{-1}
μ_{edeg}	0.0525 s^{-1}	0.0525 s^{-1}	0.0525 s^{-1}
μ_{pdeg}	0.0175 s^{-1}	0.0175 s^{-1}	0.0175 s^{-1}
α_1	0.0035 s^{-1}	0.0035 s^{-1}	0.0035 s^{-1}
α_2	0.035 s^{-1}	0.035 s^{-1}	0.035 s^{-1}

Reaction rates for TGF-β1 activation model. Parameters in the second column are the same as described in [1]. Red colored parameters are modified parameters to reflect the calcium effects. Blue colored parameters are modified parameters to reflect the KLF2 effects (kp1 reduced by 7.8 fold, kp2 reduced by 7.4 fold).
