# Supplementary material for: Computational analysis reveals the coupling between bistability and the sign of a feedback loop in a TGF-β1 activation model
Source: BMC Syst Biol. 2017 Dec 21;11(Suppl 7):136. doi: 10.1186/s12918-017-0508-z (PMC5763301; doi:10.1186/s12918-017-0508-z)
Supplement: Supplementary file 4 — List of primer sequences for genes probed on quantitative real time PCR. (PDF 26 kb) [file 12918_2017_508_MOESM4_ESM.pdf]

TABLE S3 List of primer sequences for genes probed on quantitative real time PCR

Gene name	Primer sequences (5’-3’)
β-actin	
Sense	ACCCACACTGTGCCCATCTA
Antisense	GCCACAGGATTCCATACCCA
PAI1	
Sense	TGGTGAACGCCCTCTATTTC
Antisense	GAGGGGCACATCTTTTTCAA
